# 23.1 UNDERSTANDING THE ROLE OF SCHIZOPHRENIA/AUTISM GENES IN CORTICAL DEVELOPMENT

**DOI:** 10.1093/schbul/sby014.091

**Published:** 2018-04-01

**Authors:** Helen Cooper, Amanda White, Conor O’Leary

**Affiliations:** 1 Queensland Brain Institute, The University of Queensland; 2 Queensland Brain Institute

## Abstract

**Background:**

The fidelity of neocortical development is dependent on the highly polarized morphology of the neuroepithelial stem cell (NSC) within the embryonic brain. NSCs project long processes to the pial surface along which newborn neurons migrate to establish the cortical plate. Perturbation of NSC morphology prevents neuronal migration into the emerging cortical layers, leading to cortical malformations. Disruption of the laminar architecture due to failed neuronal migration is thought to contribute to the etiology of schizophrenia and autism. Therefore, elucidating the signaling events that precisely control NSC morphology is essential to our understanding of corticogenesis and the aberrant processes that contribute to neuropsychiatric disorders.

Maintenance of NSC morphology and function requires the formation of cadherin-based cell-cell adhesion (adherens junctions) between NSCs and loss of junctional integrity results in failed neuronal migration. Junctional stability is critically dependent on the closely apposed actin cytoskeleton and the actin remodeling protein Cyfip1 known to promote actin polymerization. Cyfip1 has been implicated in schizophrenia and autism and its loss results in cortical malformations. However, the molecular mechanisms governing Cyfip1 activity in NSCs are poorly understood.

**Methods:**

In this study we investigate the signaling mechanisms that regulate Cyfip1 activity in the developing mouse cortex using both gain- and loss-of-function approaches. Short interfering RNAs or cDNA expression constructs were electroporated, in utero, into the embryonic day 12 mouse cortex. Phenotypic analysis was then performed several days later.

**Results:**

Here we identify the netrin/RGM receptor, Neogenin, as a direct binding partner for Cyfip1. We provide evidence that Neogenin is a critical upstream regulator of Cyfip1 activity during corticogenesis and is therefore a key component of NSC junctions. We show that blocking Neogenin/Cyfip1 interactions in the embryonic mouse cortex results in NSC junctional collapse and severe perturbation of the emerging cortical architecture due to aberrant neuronal migration. Our study therefore reveals that Neogenin’s interaction with Cyfip1 is essential for NSC morphology and function.

**Discussion:**

In conclusion, we have identified a novel signaling pathway that governs the development of the neocortex. The emergence of neuronal migration defects and cortical malformations when Neogenin-Cyfip1 interactions are prevented emphasizes the fundamental role of this interaction in establishing the correct cortical architecture. Intriguingly, mutations in the Neogenin gene have recently been linked to autism. Therefore, our study implicates the Neogenin/Cyfip pathway in the etiology of neuropsychiatric disorders.

